# Axitinib Induced Recurrent Pneumothorax following Near-Complete Response of Renal Cell Carcinoma Lung Metastasis: An Unexpected Complication

**DOI:** 10.1155/2012/390702

**Published:** 2012-12-26

**Authors:** Francisco Socola, Arturo Loaiza-Bonilla, Pasquale Benedetto

**Affiliations:** Division of Hematology and Oncology, Department of Medicine, Jackson Memorial Hospital and Sylvester Comprehensive Cancer Center, University of Miami Miller School of Medicine, Suite 3300, 1475 Northwest 12th Avenue, Miami, FL 33136, USA

## Abstract

We report a case of a Caucasian male with a history of renal cell carcinoma metastatic (mRCC) to the lungs refractory despite aggressive treatment with several lines of targeted therapy. He was started on axitinib palliative targeted therapy with a good clinical and radiological response; however one month after treatment initiation he presented to the emergency department with severe dyspnea and hypoxemia. Physical exam and chest X-ray revealed left-sided tension pneumothorax which required emergent thoracostomy with subsequent improvement; however it recurred requiring video assisted thoracoscopy. A left-sided 4 × 3 cm cavitated necrotic lesion was found at the level of the main pulmonary artery. Repair with pericardial fat flap was performed. Surgical biopsies from this lesion revealed mRCC with extensive necrosis. Imaging studies before and after axitinib use showed an initial 4 × 3 cm mass seen in the same location of this large cavitated necrotic tumor. Pneumothorax has not been described as a potential major complication from the use of axitinib. Complete or near-complete responses of mRCC to axitinib targeted therapy may lead to this potential life-threatening complication, particularly if the metastatic lesions are located near to pleural structures. We also review pertinent clinical trial data on axitinib.

## 1. Case Presentation

We report the case of a 56-years-old Caucasian male a histoty of clear cell renal carcinoma status post right nephrectomy 2 years prior, and who subsequently developed lung metastasis. The patient had progression of disease after several lines of treatment including to sutent, pazopanib and temsirolimus, and he was ultimately started on axitinib 5 mg by mouth twice a day with good initial clinical and radiological response after 1 one month of treatment.He presented to the emergency department of our institution complaining of 3-day-progressive dyspnea on minimal exertion, associated to dry cough and difficulty to speak. Vital signs on admission revealed a blood pressure of 93/59 mmHg, tachycardia, tachypnea, and hypoxemia with oxygen saturation of 90% on room air. Chest examination showed absent breath sounds in the left side of his chest. Chest X-ray (CXR) revealed a left-sided tension pneumothorax, which required thoracostomy by cardiothoracic surgery in an emergent fashion; a new CXR showed complete resolution of left pneumothorax, and for that reason after 3 days the chest tube was removed and the patient was discharged home. Unfortunately the next day he returned to the emergency department with a new onset of shortness of breath and left-sided chest pain; a new CXR showed left recurrent pneumothorax; thus chest tube was inserted again. High resolution CT scan showed biapical bullae and blebs, a small left pneumothorax, subcutaneous emphysema, and a 4.3 × 2.6 cm pneumatocele within the anterior segment of the left upper lobe. 

**Figure 1:**
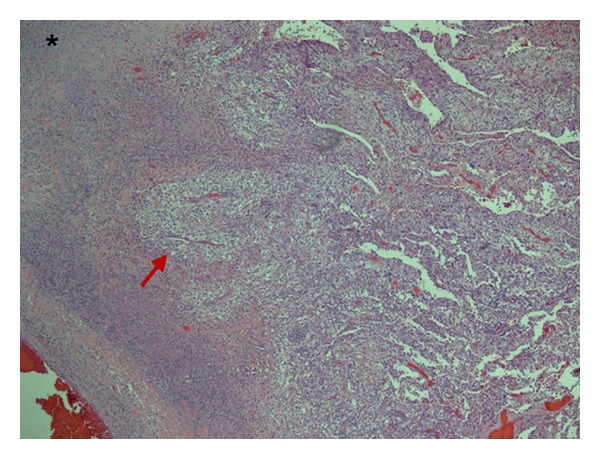
Low power microphotograph (10x) shows pulmonary parenchymal involvement by metastatic clear cell renal cell carcinoma (red arrow), adjacent to an area of extensive necrosis (asterisk).

**Figure 2:**
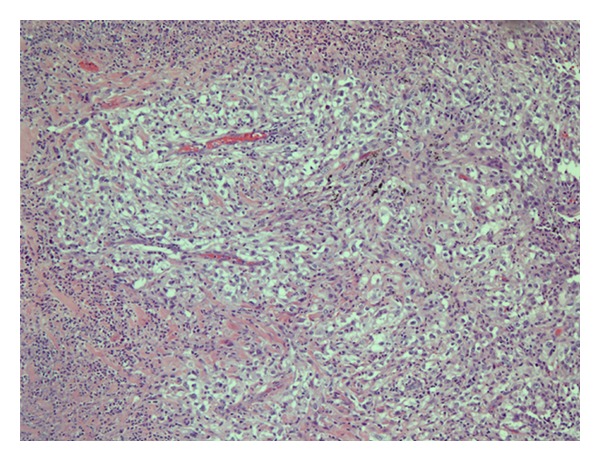
A high power view (40x) of pulmonary involvement by metastatic clear cell renal cell carcinoma. The carcinoma is composed of cells with clear cytoplasm and irregular dark nucleus with nucleoli (high nuclear grade), arranged in a solid pattern and displaying abundant fine vasculature. This tumor was morphologically identical to a previous lung wedge resection that showed metastatic clear cell renal cell carcinoma that was positive for both RCA and CD10 by immunohistochemistry.

**Figure 3:**
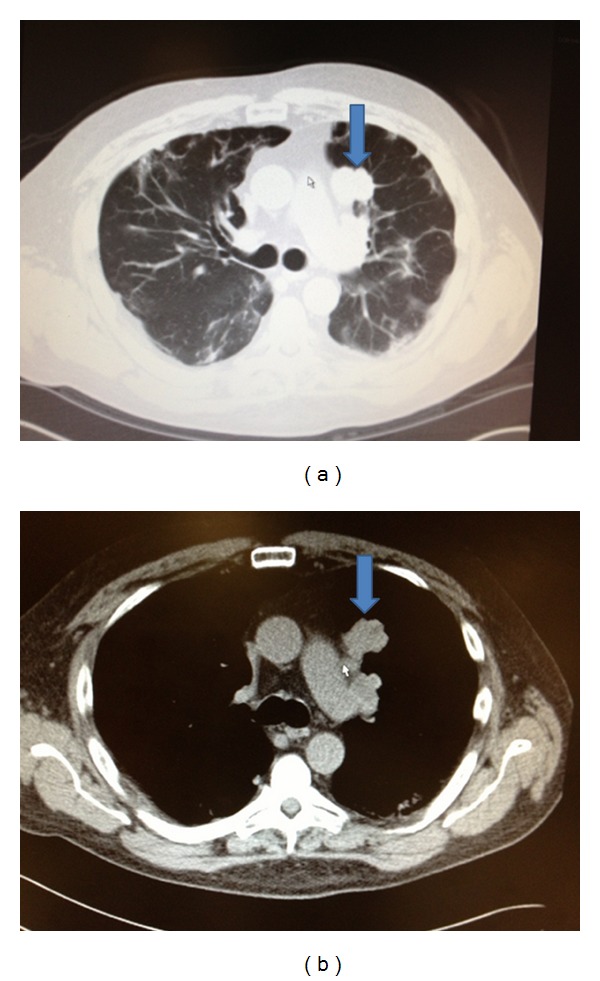
Chest CT with contrast that showed RCC metastasis to the left lung (blue arrow) in lung and mediastinum window.

**Figure 4:**
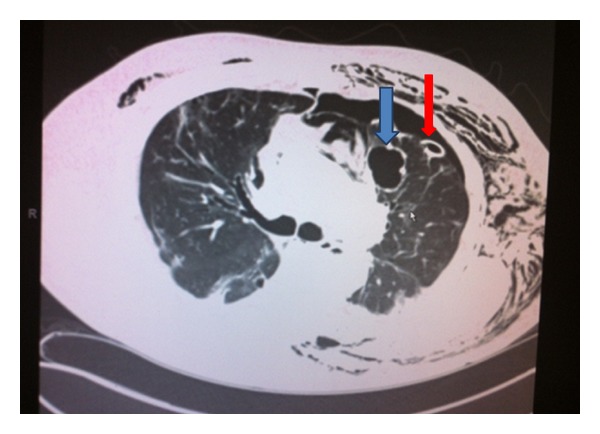
Chest CT with contrast after one month of axitinib that showed left pneumothorax, cutaneous emphysema, thoracic tube (red arrow), and necrotic RCC metastasis in the left lung (blue arrow).

Cardiothoracic surgery decided to perform a left video assisted thoracoscopy with apical blebs resection as a definitive treatment. In the operation room, and after performing a left upper lobe wedge resection of the blebs, reexpansion of the left lung was achieved and it revealed a large left upper lobe air leaking caused by 4 × 3 cm cavitated necrotic tumor at the level of the main pulmonary artery, so the decision was made to proceed with a left thoracotomy. During the procedure it was found that the necrotic mass was not resectable, and for that reason the lesion was covered and repaired via pericardial fat flap placement. Multiple biopsies were taken from this mass, with a final pathology report of a lesion consistent with mRCC with extensive associated necrosis (Figures 1 and 2). Postoperative course was also complicated with a left side empyema and new onset atrial fibrillation that were successfully treated and the patient eventually was discharged home. A comparative review of interval imaging studies before- and afteraxitinib use showed an initial 4 × 3 cm mass seen in the same location of this large cavitated necrotic tumor (Figures 3 and 4). Apparently this mass transformed into a cavitated tumor after one month of axitinib treatment and caused the recurrent pneumothorax. 

## 2. Discussion

### 2.1. Background and Pathophysiology of RCC

Renal cell carcinoma (RCC) is the most common form of kidney cancer, [1] accounting for 2-3% of adult malignancies worldwide [2] and increasing at a rate of 2-3% per decade [1]. Approximately 30% of RCC patients have metastatic disease (mRCC) at the time of diagnosis [1, 3–5]. In 2008, the incidence rate (per 100,000 persons at risk) for kidney cancer was 17.7, 13.6, and 2.0 in North America. 

The first step of treatment is the surgical removal of primary tumor [2]; however if the patient has mRCC systemic therapy is recommended. Furthermore, mRCC is resistant to traditional cancer treatments, for example, chemotherapy and radiation. Until recently, cytokine treatment with interferon-*α* (IFN-*α*) and/or interleukin-2 (IL-2) was the standard of care for mRCC [4, 6]. However, these therapies offer modest clinical benefit [4]. 

Renal cell carcinoma (RCC) is characterized by hypervascularized tumors. Angiogenesis in RCC is required for growth of tumors greater than 1 to 2 mm and is largely caused by inactivation of the von Hippel-Lindau (VHL) tumor suppressor gene and subsequent upregulation of hypoxia-inducible factor (HIF) [7]. Dysregulation of HIF plays a major role in the pathogenesis of RCC [7, 8]. Activation of mammalian target of rapamycin (mTOR) signaling can upregulate HIF expression, leading to activation of vascular endothelial growth factor (VEGF), which is a potent mediator of angiogenesis. HIF also activates platelet-derived growth factor (PDGF) and Transforming growth factor alpha (TGF-*α*) production, which are key factors in angiogenesis and tumor progression.

These factors have their respective receptors on the surface of target cells [9]; they are called vascular endothelial growth factor receptors (VEGFR), and three different ones of them have been described, VEGFR-1, 2, and 3 [10]. The interaction between the ligand and the receptor triggers autophosphorylation and initiates a series of downstream signaling processes that promote proliferation, migration, and survival of endothelial cells. In tumor vascularization, these processes form the framework of immature new neoplastic vessels [11]. Studies involving anti-VEGF receptor therapies have demonstrated that these agents can potently inhibit angiogenesis and tumor growth in preclinical models [11].

Nowadays clinical guidelines in the United States and Europe recommend sequential therapy with targeted therapies as a first line of treatment for patients with metastatic renal cell carcinoma (mRCC). Most patients are initially treated with vascular endothelial growth factor receptor-tyrosine kinase inhibitor (VEGFR-TKI), sunitinib, sorafenib, and pazopanib; however after a certain period of time they will eventually develop resistance and subsequent disease progression. For these patients the second line of treatment is a mammalian target of rapamycin (mTOR) inhibitor (e.g., everolimus and temsirolimus), which has a different mechanism of action [12]. Positive results of the phase III AXIS trial led to recent approval in the United States of the axitinib (a VEGFR-TKI) as a category 1 recommendation for use in patients with mRCC who failed one previous systemic therapy [13]. These results as well as a review of the indications, efficacy, and side effects of axitinib in clinical trials will be described in the following paragraphs. 

### 2.2. Axitinib

Axitinib (INLYTA) is a tyrosine kinase inhibitor, that inhibits multiple targets, including VEGFR-1, VEGFR-2, VEGFR-3, platelet derived growth factor receptor (PDGFR), and cKIT (CD117) [14].

Given that axitinib is more potent and selective against the VEGFR family compared with sorafenib and sunitinib, it was hypothesized that axitinib may provide clinical benefit in patients who had received prior VEGF-targeted therapy [15].

This drug has been successful in trials with renal cell carcinoma (RCC) and several other tumor types. On January 27, 2012, the US Food and Drug Administration (FDA) approved axitinib for use in patients with metastatic renal cell carcinoma that had failed to respond to a previous treatment [16].

### 2.3. Dose, Pharmacokinetics, and Drug Interactions

In the phase I, multicenter clinical trial of axitinib in patients (*n* = 36) with refractory tumors, the maximum tolerated dose for further phase II clinical trials was established as 5 mg bid [17]. The plasma elimination half-life ranges between 2 and 5 hours [18]. 

Axitinib is metabolized primarily by CYP3A4/5; concurrent use with a strong CYP3A4/5 inhibitor or inducer is not recommended. It should be reduced by about 50% in patients with moderate hepatic impairment or in those taking a strong CYP3A4/5 inhibitor [17].

### 2.4. Efficacy

The AXIS trial was a phase 3 randomized trial that showed the efficacy of this drug. In this study it was showed that axitinib may increase progression-free survival (PFS) compared to sorafenib in patients with metastatic renal cell carcinoma who previously had failed to first-line therapy with sunitinib, bevacizumab plus interferon-alfa, temsirolimus, or cytokines. 389 patients (54%) had received 1 prior sunitinib-based therapy, 251 patients (35%) had received 1 prior cytokine-based therapy (interleukin-2 or interferon-alfa), 59 patients (8%) had received 1 prior bevacizumab-based therapy, and 24 patients (3%) had received 1 prior temsirolimus-based therapy. The baseline demographic and disease characteristics were similar between the axitinib and sorafenib groups with regard to age (median 61 years), gender (72% male), race (75% white and 21% Asian), and Eastern Cooperative Oncology Group (ECOG) performance status (55% 0, 45% 1), and histology (99% clear cell) [19].

In this study they recruited 723 patients (median age 61 years) with metastatic renal cell carcinoma; they were randomized (1 : 1) without blinding to axitinib 5 mg (*N* = 361) twice daily versus sorafenib 400 mg (*N* = 362) twice daily; allocation concealment was not done. All patients had renal clear-cell carcinoma that progressed despite first-line therapy with sunitinib, bevacizumab plus interferon-alfa, temsirolimus, or cytokines. Increased Axitinib dose was allowed for patients without hypertension or adverse reactions [19].

Progression free survival (PFS) was defined as time from randomization to either first documentation of disease progression (per independent blinded radiology review of images) or death due to any cause. The median duration of axitinib therapy was 6.4 months and sorafenib therapy was 5 months. Disease progression or relapse occurred in 160 patients (44%) in axitinib group and 180 patients (50%) in sorafenib group. There was a statistically significant advantage for axitinib over sorafenib for the endpoint of PFS. There was no statistically significant difference between the arms in OS. Comparing axitinib versus sorafenib in intention-to-treat analysis, the PFS was 6.7 months in the axitinib group versus 4.7 months in the sunitinib group; hazard ratio was 0.665 (95% CI 0.544–0.812); one-sided *P* < 0.0001, and the objective tumor response was 19% versus 9% respectively (*P* = 0.0001, NNT 10) [19].

### 2.5. Side Effects

In the AXIS trial the most frequent adverse events associated with axitinib were diarrhea, hypertension, fatigue, decreased appetite, nausea, and dysphonia, each occurring in more than 30% of patients. Hypertension, nausea, dysphonia, and hypothyroidism were more common with axitinib as well [19].

To evaluate axitinib side effects a single-stage, open-label, multicenter, phase II study was done, which recruited 62 patients, 100% had received prior sorafenib, and 74.2% had received two or more prior systemic treatments; they received axitinib 5 mg orally twice daily. The most common side effects were mild (grade 1 and 2); they included fatigue, diarrhea, anorexia, hypertension, and nausea. The most common grade 3 AEs were hand-foot syndrome (16.1%), fatigue (16.1%), hypertension (16.1%), and diarrhea (14.5%). There were no treatment-related deaths. Two patients developed congestive heart failure (CHF). Two patients had cerebral hemorrhage, in the first one, who had received 32 days of axitinib, a subsequent scan revealed a previously undetected brain metastasis at the hemorrhage site. The second experienced elevated BP after 2 days of axitinib and developed grade 4 cerebral bleeding on day 5; a contralateral brain mass was observed, and axitinib was discontinued. There was one patient that developed a gastrointestinal (bowel) perforation; however this patients also was receiving radiation therapy in the abdomen. Eighteen patients (29.0%) received levothyroxine for suspected hypothyroidism during the study. Most hematologic AEs were mild to moderate, and there were no grade 4 hematologic events [15].

Finally in one axitinib phase I study, two nonsmall cell lung cancer (NSCLC) patients exhibited lung cavitations indicating an antiangiogenic effect [17, 18]. The development of pneumothorax associated to the use of axitinib was suggested in December 7, 2011 ODAC Briefing Document for axitinib (NDA 202324), where it was briefly reported that 4 (1.1%) of the 359 RCC patients that received axitinib suffered nonfatal pneumothoraces (compared to only 1 of 355 RCC patients receiving sorafenib) [20].

## 3. Conclusion

We report the case of a male with pulmonary metastases of renal cell carcinoma and subsequent transformation of one of these lesions into a cavitated necrotic tumor after axitinib treatment, which caused recurrent pneumothorax. Apparently axitinib, the most potent and new VEGFR inhibitor, led to central necrosis of the tumor which induced to a direct communication between the bronchial airway and the pleural cavity, causing the pneumothorax. To the best of our knowledge, this is the first reported case of recurrent pneumothorax as a major complication of axitinib treatment in a patient with metastatic renal cell carcinoma. Our case has several particular characteristics including a difficult-to-diagnose underlying cause of recurrent pneumothorax, the rarity of this axitinib complication, and the required surgical treatment of this condition. We believe that it is very important to publish this case report to increase awareness of this rare but life-threatening complication, especially in patients who have multiple, large metastases located close to the visceral pleura. In addition, the advent of potential new indications for axitinib treatment may lead to an increase incidence of this severe adverse event thus the need for surveillance. The pathophysiology and infrequency with which this axitinib complication is encountered make it a formidable diagnostic and therapeutic challenge.
